# DEFA5-producing CD4^+^ T cells in the intestines of atopic dermatitis patients play an important role in the development of AD-associated intestinal inflammation

**DOI:** 10.3389/fimmu.2025.1535527

**Published:** 2025-09-19

**Authors:** Kai Zhuang, Mengjun Li, Yalan Wu, Yi Luo, Jian Song, Sze Chun Leo Chan, Jinmei Li, Ziying Chen, Yulin Ouyang, Yongliang Zhang, Ying Lin, Huanhuan Luo

**Affiliations:** ^1^ State Key Laboratory of Traditional Chinese Medicine Syndrome, Guangzhou University of Chinese Medicine, Guangzhou, China; ^2^ Chinese Medicine Guangdong Laboratory, Hengqin, China; ^3^ School of Basic Medical Sciences, Guangzhou University of Chinese Medicine, Guangzhou, China; ^4^ Department of Microbiology and Immunology, and Immunology Translational Research Programme, Yong Loo Lin School of Medicine, National University of Singapore, Singapore, Singapore; ^5^ Immunology Programme, The Life Science Institute, National University of Singapore, Singapore, Singapore; ^6^ School of Pharmaceutics, Guangzhou University of Chinese Medicine, Guangzhou, China; ^7^ Department of Dermatology, The Second Affiliated Hospital of Guangzhou University of Chinese Medicine, Guangzhou, China; ^8^ Guangdong Provincial Key Laboratory of Chinese Medicine for Prevention and Treatment of Refractory Chronic Diseases, Guangzhou, China; ^9^ Guangdong Provincial Clinical Research Center for Chinese Medicine Dermatology, Guangzhou, China

**Keywords:** atopic dermatitis, single-cell RNA sequencing, intraepithelial lymphocytes, DEFA5, PPARγ

## Abstract

**Rationale:**

Atopic dermatitis (AD) is associated with various gastrointestinal symptoms, yet the underlying mechanisms remain poorly understood. This study aimed to investigate intraepithelial lymphocytes (IELs) in the intestines of AD patients and their potential contribution to intestinal inflammation.

**Methods:**

Single-cell RNA sequencing was utilized to analyze the immune cell composition in the ileum of adult AD patients with severe symptoms. Laser confocal microscopy, Western blot, polymerase chain reaction and adoptive T cell transfer experiments were carried out to investigate the phenotypes of IELs and contribution of CD4^+^ IELs in intestinal inflammation and barrier function.

**Results:**

A distinct group of DEFA5-expressing CD4^+^ T cells in the small intestine of AD patients was identified. These cells were enriched in tissue resident memory T cells (Trm). Peroxisome proliferator-activated receptor (PPAR) was found to be important for the function of DEFA5-expression CD4^+^ IELs. In an AD mouse model, an increase in DEFA5-expressing CD4^+^ IELs was observed compared to control, and these cells contributed to the development of intestinal inflammation and impaired intestinal barrier function.

**Conclusions:**

AD is linked to an increase in intestinal DEFA5-expressing CD4^+^ IELs, which may play an important role in mediating intestinal inflammation. This suggests that the DEFA5-expressing CD4^+^ IELs could be a potential therapeutic target for managing gastrointestinal symptoms in AD patients.

## Graphical Abstract

A graphical scheme illustrating the ability of CD4^+^ IELs in the small intestine of ADs to produce DEFA5, thereby inducing inflammatory lesions in the intestines. IELs, intraepithelial lymphocytes; ADs, atopic dermatitis patients; DEFA5, defensin alpha 5.The abstract should ideally be structured according to the IMRaD format (Introduction, Methods, Results and Discussion). Provide a structured abstract if possible. If your article has been copyedited by us, please provide the updated abstract based on this version.

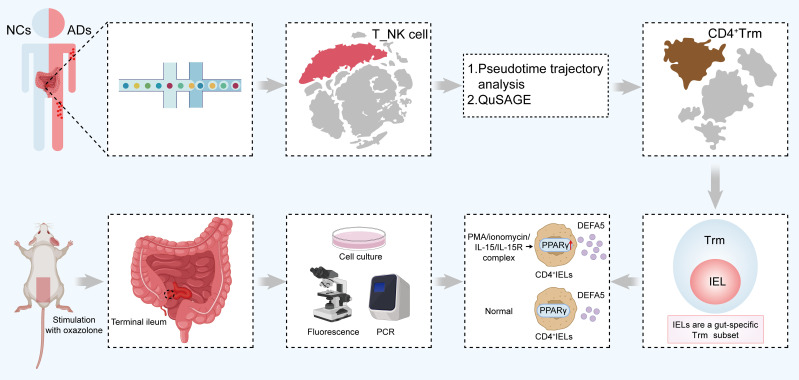

## Introduction

Host defense peptides, evolutionarily conserved molecules in the innate immune system, exhibit both direct antimicrobial activity and immune modulatory function in a broad range of organisms. In mammals, these peptides are predominantly categorized into defensins and cathelicidins. Defensins are subcategorized into α-, β-, and θ-types, with only α- and β-defensins confirmed to exist in humans (1). Thus far, six α-defensins have been identified in humans, categorized into myeloid and enteric α-defensins according to variations in their encoding exons (2). Among α-defensins, defensin alpha 5 (DEFA5) was initially identified as a Paneth cell-specific peptide that was confined to the base of intestinal crypts (3). Subsequent research revealed that DEFA5 can also be detected in the mucosal epithelia of other tissues, such as the reproductive and respiratory tracts (4, 5). In addition to its antimicrobial properties, DEFA5 may be exploited by bacteria such as *Shigella* to promote their adhesion and invasion (6, 7). Additionally, DEFA5 has potent chemotactic effects on various immune cells including macrophages, mast cells, as well as naïve and memory T lymphocytes (8). Furthermore, it induces the expression of IL2, IL8 and IFNγ in CD4^+^ T cells (9), suggesting that it could regulate intestinal inflammation through modulation of the recruitment and function of immune cells. In the healthy small intestine, the ratio of intraepithelial lymphocytes (IELs) to epithelial cells is approximately 10 to 20 per 100, indicating that IELs are among the main lymphocytic contingents within the intestinal immune milieu (10). Furthermore, IELs are considered as a major constituent of intestinal tissue-resident memory T (Trm) cells (11). Previous studies have demonstrated a significant increase in intestinal mucosal CD4^+^ Trm cells in patients with inflammatory bowel disease, where they contribute to proinflammatory responses (12). CD4^+^ IELs are categorized as part of the induced IELs based on their developmental lineage and phenotypic attributes. These induced IELs are reactive to antigens encountered in the periphery and subsequently relocate to the intestinal epithelial layer, where they express activation markers, specifically CD69 and CD25 (13–15). Additionally, studies have shown that CD8^+^ IELs can directly produce various α-defensins, a process that is dependent on the activation of Toll-like receptors (16).

Accumulating evidence have illuminated reciprocal interactions between gastrointestinal and dermatological disorders (17, 18). Atopic dermatitis (AD) is a chronic, immune-mediated inflammatory skin disorder, and epidemiological data indicate that its prevalence is approximately 2.1–4.9% in adults (19). Notably, over 50% of pediatric patients exhibit a propensity for recurrence in adulthood (20). Recent findings revealed that approximately 28.6% and 24.1% of adult patients with AD exhibit concomitant food sensitivities and allergic reactions, respectively, associated with gastrointestinal inflammation (21), suggesting the bidirectional relationships between dermatological conditions and gastrointestinal pathologies. However, the precise molecular mechanisms underlying these associations between dermatological conditions and gastrointestinal pathologies have yet to be fully elucidated (22, 23). Interestingly, elevated levels of α-defensins in individuals afflicted with AD have been described (24). However, the sources of α-defensins and their contribution to AD-associated intestinal inflammation are unclear.

In this study, using a high-resolution single-cell type map of the ileal mucosa in adult patients with AD, we identified DEFA5-producing CD4^+^ IELs which may play an important role in the development of AD-associated intestinal inflammation.

## Materials and methods

### Patient cohorts

In this study, we enrolled 5 patients with AD and 5 healthy volunteers who received treatment at the Guangdong Provincial Hospital of Chinese Medicine between December 2021 and July 2022. Ethical approval for this research was obtained from the Ethics Committee of the Second Affiliated Hospital of Guangzhou University of Chinese Medicine, as indicated by Approval ID BF2021-220-01. Prior to participation, all individuals involved in the study provided written informed consent, ensuring adherence to ethical research standards. Comprehensive clinical information for all patients is detailed in **Supplementary Table S1**.

### Preparation of single-cell suspensions

The samples were processed as follows: Firstly, the tissue was washed with precooled phosphate-buffered saline (PBS, #C10010500BT, Gibco, Carlsbad, USA) and immediately sectioned into 1 mm³ fragments on ice. These fragments were subjected to enzymatic digestion at 37°C using a mixture of 0.5 U/mL dispase II (#LS02109, Worthington, Lakewood, USA), 50 U/mL DNase I (#LS002007, Worthington), 285 U/mL collagenase I (#LS004196, Worthington), and 355 U/mL collagenase II (#LS02109, Worthington) with gentle agitation for 45 minutes. Following digestion, the samples were passed through a 70 µm cell strainer and then centrifuged at 300 × g for five minutes. The resulting cell pellet was then suspended in red blood cell lysis solution (#130094183, Miltenyi Biotec, Shanghai, China) to remove any remaining erythrocytes, followed by washing with PBS containing 0.04% BSA and resuspension in the same buffer. To ensure the isolation of single cells, the cell suspension was further filtered through a 35 μm cell strainer. Finally, the isolated single cells were stained with acridine orange/propidium iodide (AO/PI), and their viability was assessed using a Countstar fluorescence cell analyzer, ensuring the preparation of high-quality single-cell suspensions for subsequent analyses.

### Single-cell RNA sequencing

To generate the scRNA-Seq libraries, we utilized the 10× Genomics Chromium Controller Instrument and Chromium Single Cell 3’V3 Reagent Kits (10× Genomics, Pleasanton, USA). In this process, the cells were adjusted to a concentration of 1000 cells/μL. We then loaded approximately 8,000 cells into each channel of the instrument, aiming to barcode approximately 5,000 single cells per sample within the gel bead-in-emulsion (GEM) system. Following reverse transcription, the GEMs were broken down, and the barcoded cDNA was isolated. This cDNA then underwent a series of processing steps: it was purified, amplified, and subjected to fragmentation and A-tailing. The final library quality was assessed using the Qubit high-sensitivity DNA assay (Thermo Fisher Scientific, Waltham, USA), and the size distribution was analyzed with a Bioanalyzer 2200 (Agilent, Santa Clara, USA) high-sensitivity DNA chip. Sequencing was performed on an Illumina sequencer (Illumina, San Diego, USA) with a 150 bp paired-end run, ensuring a depth of 50,000 reads per cell.

### Single-cell RNA statistical analysis

Single-cell RNA-seq data analysis was conducted using the NovelBrain Cloud Analysis Platform provided by NovelBio Co., Ltd. (www.novelbrain.com). The procedure commenced with the utilization of the fastp tool (25), set to its default parameters, for the purpose of filtering out adaptor sequences and eliminating low-quality reads, thereby yielding data of enhanced quality. Subsequently, the data were aligned to the human genome reference GRCh38 (Ensembl104) using CellRanger v6.1.1, thereby generating feature-barcode matrices. To ensure uniformity across all sequenced samples, a downsampling analysis based on the number of mapped barcoded reads per cell was performed, which led to the formation of a final aggregated matrix. Only cells expressing more than 200 genes and displaying mitochondrial UMI rates less than 70% were deemed high quality and retained. After the removal of mitochondrial genes, the expression data were normalized and regressed using the Seurat package (version 4.0.3, https://satijalab.org/seurat/), accounting for UMI counts and mitochondrial rates to produce scaled data. Principal component analysis of these data was carried out, concentrating on the 2000 most variably expressed genes, with the top 10 principal components utilized for tSNE and UMAP plotting. A graph-based clustering method was applied for unsupervised cell clustering, drawing on these principal components. Marker genes were identified through the FindAllMarkers function using the Wilcoxon rank sum test under the following criteria: lnFC > 0.25, p value < 0.05, and min.pct > 0.1. For in-depth identification of diverse cell types, selected cell clusters were subjected to further reanalysis involving retSNE, graph-based clustering and marker gene assessment.

### Animals

Six-week-old male BALB/c mice were procured from Zhuhai BesTest (license number: SCXK 2020-0051). These animals were maintained in a controlled environment characterized by a temperature of 23 ± 3°C and relative humidity (RH) of 55 ± 5% under a 12-hour light/dark cycle. The mice had ad libitum access to standard laboratory food and water. All breeding activities were conducted at the Research Centre of Basic Integrative Medicine, School of Basic Medical Sciences, Guangzhou University of Chinese Medicine (license number: SYXK 2023-0182). The animal experiments conducted in this study were in strict compliance with the relevant regulations pertaining to the ethics of experimental animal use. Ethical approval for these experiments was granted by the Animal Experiment Management and Use Committee of Guangzhou University of Chinese Medicine, as indicated by the experimental approval reference number 20230615027.

### Oxazolone-induced AD in BALB/c mice

The AD mouse model was adapted from previous methods with modifications (26–28). After a week-long acclimation phase, the mice were methodically segregated into two groups, each comprising eight groups: (1) a control group without oxazolone (#862207, Sigma–Aldrich, Saint Louis, USA) and (2) a model group with 1% oxazolone. On the initial day of the experiment, the mice were anesthetized with 2% isoflurane, and a dorsal section measuring 3.5 cm × 3.5 cm was carefully shaved. A sensitizing mixture containing 3% oxazolone in 100 μL of vehicle solution (consisting of acetone and olive oil at a 4:1 ratio) was applied to the hairless dorsal area for 7 days. The control group was administered 100 μL of vehicle. On the eighth day, 10 μL of 1% oxazolone solution was applied to both sides of the left ear, initiating a series of seven challenges. On the final day of application (Day 14), the mice were euthanized. The ear and intestinal tissues were then excised for subsequent experimental analysis.

### Flow cytometry

For surface staining, IELs and spleen lymphocytes (SPLs) were initially treated with an Fc receptor blocking reagent and then incubated with specific antibodies for 30 minutes at 4°C. For intracellular staining, the cells were subjected to a 5-hour pretreatment with phorbol myristate acetate (PMA, 50 ng/mL, #P8139, Sigma–Aldrich), ionomycin (1 µg/mL, #I3909, Sigma–Aldrich) and Brefeldin A (5 µg/mL, #B7651, Sigma-Aldrich), followed by surface marker staining. After fixation and permeabilization, intracellular antibodies were applied. Details regarding the antibodies used are listed in **Supplementary Table S2**. All flow cytometry experiments were carried out with a flow cytometer (LSRFortessa, BD Biosciences, Franklin Lakes, USA), and the data were analyzed using FlowJo software (Tree Star, Ashland, USA).

### Isolation of IELs and SPLs

The mice were euthanized, and the intestines were promptly excised and immediately placed in precooled PBS. Subsequent to the careful removal of residual mesenteric fat tissue, Peyer’s patches were delicately dissected. The intestines were then longitudinally opened and rigorously washed in ice-cold PBS. Next, the intestinal segments were sectioned into 1.5 cm pieces. These pieces were then subjected to two incubation periods in 5 ml of 5 mM EDTA dissolved in Hank’s Balanced Salt Solution (HBSS, #PB180323, Pricella, Wuhan, China), each lasting 15–20 minutes at 37°C with gentle agitation at 100 rpm. After each incubation, the solution was subjected to vigorous vortexing and then filtered through a 70 µm cell strainer, followed by the addition of fresh EDTA solution. The supernatants obtained from IELs isolation from a single small intestine were pooled, and the cells were washed once in cold PBS. The cell suspension was then reconstituted in 10 ml of lymphocyte separation medium. This mixture was centrifuged for 20 minutes at 2500 rpm at room temperature to achieve separation. The cells were subsequently washed once more and resuspended in T-cell medium, after which they were immediately utilized for experimental purposes. The spleens were excised, immersed in cold PBS and then filtered through a 100 µm strainer. Erythrocytes were lysed using RBC lysis buffer, and the isolated SPLs were prepared for further experimentation.

### Sorting and stimulation of CD4^+^ T cells for secretion

CD4^+^ T cells were isolated using a mouse CD4^+^ T-cell cell isolation kit (#70901, Beaver, Suzhou, China) according to the manufacturer’s protocol. Once purified, the cells were cultured in RPMI-1640 medium (#PM150110A, Pricella) supplemented with 10% fetal bovine serum (FBS, #164210, Pricella) and maintained at 37°C for 24 hours. During this period, PMA (50 ng/mL, Sigma–Aldrich) and ionomycin (1 µg/mL, Sigma–Aldrich) were added for the final 6 hours of incubation, while the interleukin (IL)-15/IL-15R complex (50 ng/mL, #HY-P70655, MedChemExpress, Monmouth Junction, USA) was added during the entire 24 hours period. Afterward, the cells were centrifuged to separate the cellular components, and the supernatants were collected for subsequent analysis.

### Cell culture

Subconfluent monolayers of HT-29 cells obtained from Wuhan Pricella Biotechnology Co., Ltd. (#CL-0118, Pricella) were cultured in McCoy’s 5A medium (#PM150710, Pricella) supplemented with 10% FBS and 1% penicillin/streptomycin (#PB180120, Pricella) at 37°C in a humidified 5% CO_2_ atmosphere. DEFA5 was procured from Abmart Shanghai Co., Ltd. (#RG310219, Abmart, Shanghai, China). On the day before initiating DEFA5 treatment, the cells were subjected to starvation. HT-29 cells were then treated with DEFA5 at concentrations of 1 µg/ml and 2 µg/ml for 24 hours, after which the cells were harvested for subsequent analyses.

### Intestinal explant culture

Intestinal explants were extracted following the method previously outlined (29). In brief, the terminal ileum was sliced into 0.5 cm segments along its length, rinsed with cold PBS, and then placed into 1 mL of culture medium made up of RPMI 1640 (Thermo Fisher Scientific, Waltham, USA), protease inhibitor cocktail (Sigma-Aldrich, St. Louis, USA), and Penicillin/Streptomycin solution (Waltham, MA, USA). Afterward, the explants were co-cultured with CD4^+^ IELs (1*10^6^ cells/well), which were either stimulated or unstimulated, in 24-well plates at 37°C for 24 hours. Following the incubation period, explants from each group were harvested and preserved at -80°C for subsequent analysis.

### Adoptive transfer of CD4^+^ IELs

SCID mice were modeled for AD using the previously described method, and adoptive transfer was performed on day 7. CD4^+^ IELs were isolated from the small intestine of Balb/c mice as described in the Tissue Preparation and Cell Isolation section. After isolation, 1*10^6^ cells were injected i.p. into SCID mice. For the IELs adoptive transfer experiment, SCID mice were harvested on day 7 post-transfer.

### Western blotting

Cultured cells and animal tissue samples were subjected to three washes in ice-cold PBS, followed by lysis using RIPA buffer supplemented with protease and phosphatase inhibitors for protein extraction. The proteins extracted were separated using 10–12% SDS–PAGE and then transferred to PVDF membranes (Bio-Rad, Hercules, USA). After transfer, the membranes were blocked with 5% skim milk for 2 hours and incubated with primary antibodies at 4°C overnight. After they were washed, they were exposed to the corresponding secondary antibodies for 2 hours at room temperature. Protein band visualization was conducted with Tanon 5200 Multi (Tanon, Shanghai, China), and quantitative analysis was performed using ImageJ software. Details regarding the antibodies used are listed in **Supplementary Table S2**.

### Confocal microscopy

To investigate the colocalization of DEFA5 and CD4, CD4^+^ IELs were incubated overnight at 4°C with primary antibodies against CD4 (1:200, Proteintech, Rosemont, USA) and DEFA5 (1:100, Abbexa, Cambridge, UK) in PBS. Following primary antibody incubation, the sections were washed, and the nuclei were stained with DAPI/Hoechst (1:10000, Life Technologies, Carlsbad, USA) for 5 minutes at RT. After a final wash, confocal fluorescence microscopy was performed using a Zeiss LSM800 (Zeiss, Jena, Germany), with imaging conducted at 488 nm and 594 nm wavelengths using a 20× magnification lens.

### Real-time polymerase chain reaction

RNA was isolated from 1 million CD4^+^ IELs, CD4^+^ SPLs using TRIzol™ Reagent (#10296010, Invitrogen, Carlsbad, USA). The primer sequences synthesized by Sangon are detailed in **Supplementary Table S3** of the **Supplementary Material**.

### Statistical analyses

The data were analyzed using GraphPad Prism 9.0 software (GraphPad Software, Inc., San Diego, USA). The quantitative data represent at least three independent experiments. Unpaired Student’s t tests were utilized for comparisons between two groups. Multiple comparisons were analyzed by one-way analysis of variance (ANOVA) with Bonferroni *post hoc* correction. A p-value less than 0.05 was considered to indicate statistical significance.

## Results

### AD patients exhibit unique gene expression patterns revealed by single-cell RNA-seq analysis

**Figure 1:**
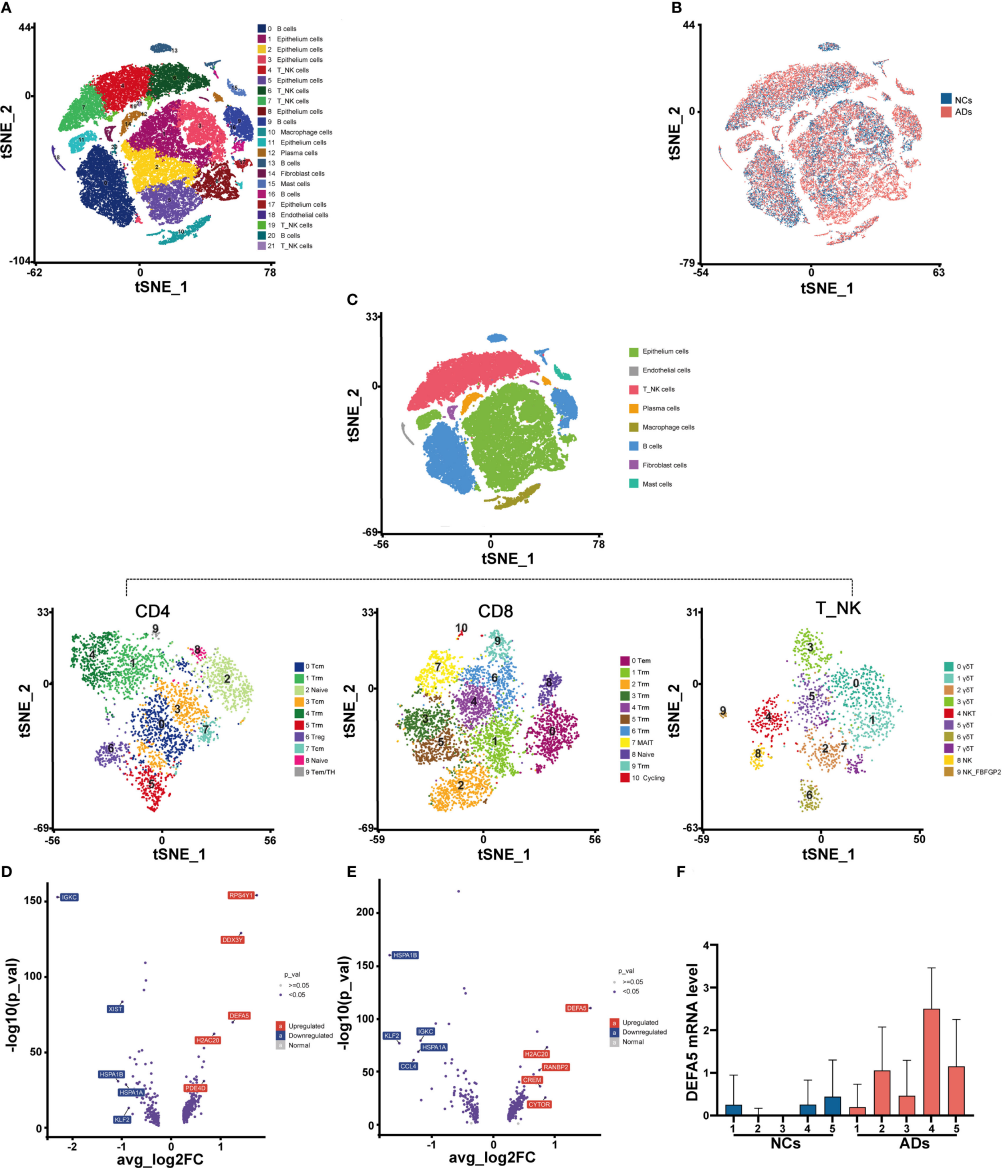
AD patients exhibit unique gene expression patterns according to a single-cell transcriptomic atlas. **(A)** t-Distributed stochastic neighbor embedding (t-SNE) facilitated the dimensional reduction of 49,971 cells, which were subsequently stratified and depicted through a color-coding scheme aligned with cell-type annotations. **(B)** Identification of cells in different groups in a tSNE plot, with cells color-coded according to sample identity: NCs in blue and ADs in red. **(C)** t-SNE of NK_T cells redefined into three subcellular groups (CD4^+^ T, CD8^+^ T, and γδT/NKT/NK cells). **(D)** Volcano plots displaying the DEGs in CD4^+^ T cells among ADs vs. NCs. Each dot represents one gene. Representative differentially expressed genes (blue) are indicated. Blue boxes, differentially downregulated genes with logFC < -0.25 and FDR < 0.05; red boxes, differentially upregulated genes with logFC > 0.25 and FDR < 0.05; blue dots, differentially expressed genes; gray dots, nondifferentially expressed genes. **(E)** Volcano plots displaying the DEGs in CD8^+^ T cells among ADs vs. NCs. **(F)** Box plot displaying the expression levels of DEFA5 in CD4^+^ T cells from individual donors.

To investigate the immune composition in the intestine of patients with AD, we collected terminal ileum tissue samples from five adult AD patients (ADs) and five normal control individuals (NCs) for single-cell RNA-seq analysis. Detailed clinical information about the cohort is provided see (**Supplementary Table S1**). Following stringent quality control measures (see **Supplementary Figures S1A, B**), we obtained a total of 75,912 cells, including 43,085 cells from ADs and 32,827 cells from normal controls for further analysis. Utilizing cluster-specific marker genes and unsupervised t-distributed stochastic neighbor embedding (t-SNE), we identified a total of 22 cell clusters (**Figure 1A**). To explore the transcriptomic heterogeneity between NCs and ADs, we delved deeper into the analysis of these 22 cell clusters. The tSNE plots revealed the cellular distribution within the NC and AD groups (**Figure 1B**). In total, five clusters, including clusters 4, 6, 7, 19, and 21 were classified as T_NK cells based on differential gene expression of *CD3D*, *CD3E* and *PTPRC* (**Supplementary Figures S1C–E**).

We further delineated the cell populations within the T_NK cell cluster through tSNE analyses. The T_NK cells identified were categorized into CD4^+^ T cells, CD8^+^ T cells, natural killer T (NKT) cells, NK cells, and γδT cells (**Figure 1C**). The NKT cells (Cluster 4), NK cells (Clusters 8 and 9), and γδT cells (Clusters 0, 1, 2, 3, 5, 6, and 7), which constituted a minor proportion, were classified into eight distinct clusters (**Figure 1C**). We further conducted differential gene expression analysis on CD4^+^ T cells (**Figure 1D**) and CD8^+^ T cells (**Figure 1E**). It was found that *DEFA5* was among the top upregulated genes in ADs compared to NCs. In light of evidence showing the ability of CD8^+^ T cells to secrete a variety of α-defensins (16), we hypothesize that CD4^+^ T cells in the gut may have similar function. Independent analyses of *DEFA5* expression in CD4^+^ T cells from the donors confirmed the increased expression of this gene in CD4^+^ T cells from ADs compared to these from NCs (**Figure 1F**).

### Expression of defensin in the intestinal CD4^+^ T cells of ADs

**Figure 2:**
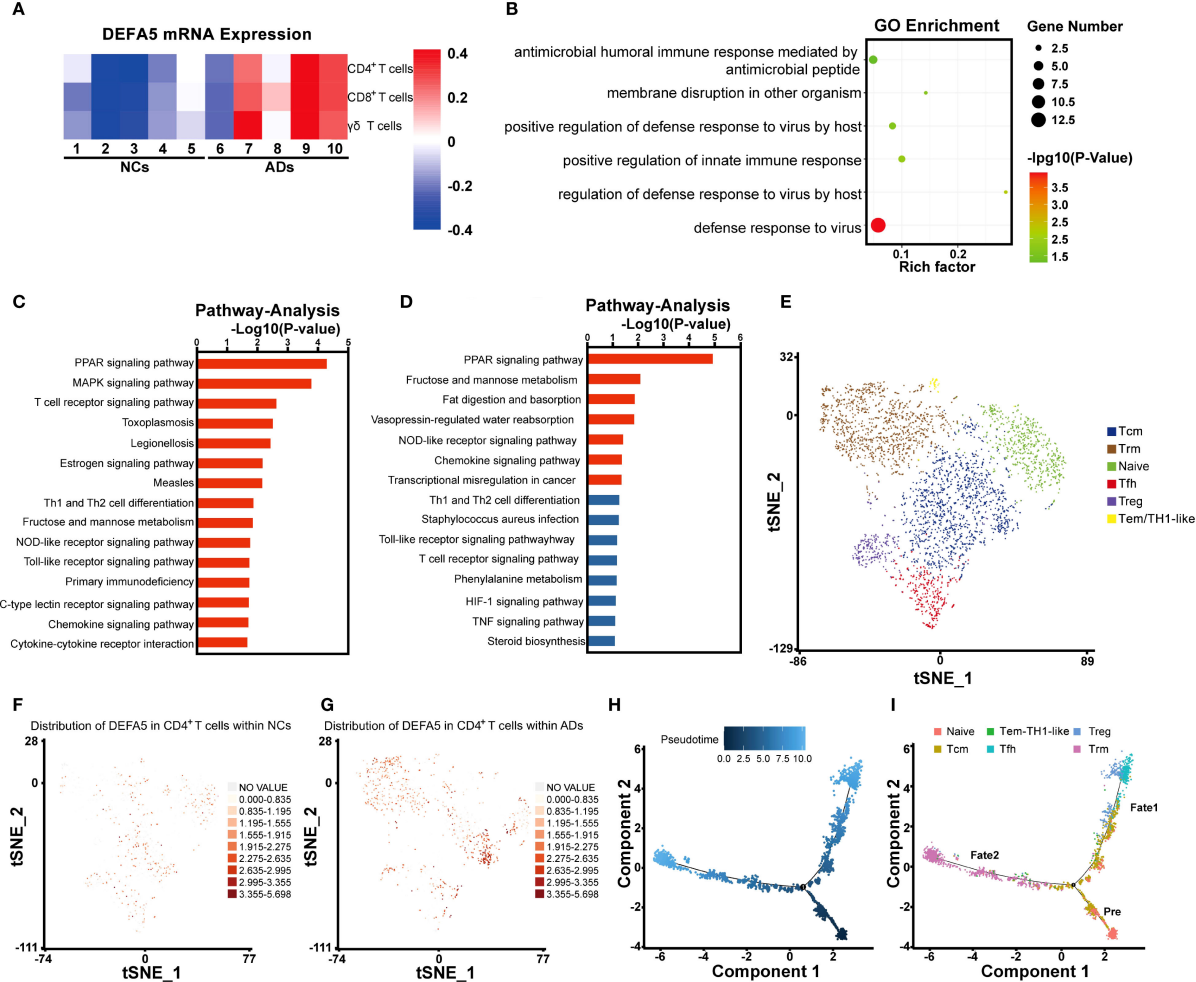
Defensin expression in the intestinal CD4^+^ T cells of atopic dermatitis patients. **(A)** Heatmap showing the expression of DEFA5 across CD4^+^ T cells, CD8^+^ T cells, and γδT cells. **(B)** Gene Ontology enrichment analysis of CD4^+^ T cells. **(C)** Pathway analysis of CD4^+^ T cells, showing the top 15 entries with the most significant changes between ADs and NCs. **(D)** Pathway analysis of CD4^+^ T cells, showing the 15 genes with the most significant upregulation between ADs and NCs. **(E)** t-SNE of CD4^+^ T cells redefined into six subcellular groups (naïve, Tfh, Tcm, Tem/TH1-like, Trm, and Treg). **(F, G)** The distribution and expression of DEFA5 in ADs and NCs are illustrated using a t-SNE plot. **(H, I)** Monocle pseudotime analysis revealing three branches: prebranch, Fate 1 and Fate 2. The distribution of single cells from each cluster mapped in a continuous lineage path.

Next, we analyzed the expression of *DEFA5* among several key cell types within the T_NK cells, including CD4^+^ T cells, CD8^+^ T cells and γδT cells, and found significantly increased expression of *DEFA5* in all types of cells in ADs compared to NCs (**Figure 2A**). Subsequent Gene Ontology enrichment analysis of CD4^+^ T cells revealed significant enrichment of defensin-associated gene sets, including “defense response to virus”, “host regulation of defense response to virus” and “membrane disruption in another organism” (**Figure 2B**). Furthermore, pathway analysis in the *DEFA5*-positive subset of CD4^+^ T cells revealed that the top differentially enriched pathways include the PPAR signaling pathway, MAPK signaling pathway, and NOD signaling pathway (**Figure 2C**). Particularly, the PPAR signaling pathway was the top upregulated pathway in the CD4^+^ T cells of ADs in comparison to NCs (**Figure 2D**).

Initially, we attempted to classify DEFA5-expressing CD4^+^ T cells into Th1 (*IFNG* ^+^, *CXCR3* ^+^, *CCR5* ^+^, *IL2* ^+^), Th2 (*IL10* ^+^, *IL4* ^+^, *IL5* ^+^, *IL13* ^+^), and Th17 (*IL17A^+^*, *IL17F^+^*, *IL22^+^*) subsets. However, DEFA5 was predominantly enriched in a previously uncharacterized cell population (**Supplementary Figures S2A, B**). To further characterize *DEFA5*-expression CD4^+^ T cells, we analyzed and identified six distinct CD4^+^ T-cell subclusters (**Figure 2E**), including CD4^+^ naïve T cells (*CCR7* ^+^, *SELL* ^+^, *LEF1* ^+^, and *TCF7* ^+^) (30), Follicular helper T (Tfh) cells (*CXCR5* ^+^, *CD200* ^+^, *TOX* ^+^, and *TOX2* ^+^) (31, 32), CD4^+^ central memory T (Tcm) cells (*CCR7* ^+^, *TCF7* ^+^, and *CD69* ^+^) (33, 34), CD4^+^ effector memory T (Tem)/TH1-like cells (*IFNG* ^+^, *CCL5* ^+^, and *GZMK* ^+^) (35, 36), CD4^+^ resident memory T (Trm) cells (*CXCR6* ^+^ and *KLRB1* ^+^) (37, 38), and Treg cells (*FOXP3* ^+^, *IKZF2* ^+^, and *CTLA4* ^+^) (39, 40). As depicted in **Figures 2F, G**, there was a notable increase in *DEFA5* expression in the intestinal CD4^+^ T cells of the ADs relative to that in the NCs. In addition, *DEFA5* expression was primarily enriched in Tcm and Trm cells. Based on the notable transcriptomic characteristics exhibited by CD4^+^ T cells, we conducted a pseudotime trajectory analysis. The results suggested that the intestinal T cells differentiated from naïve CD4^+^ T cells may diverge into two distinct evolutionary pathways: one leading toward Treg/Tfh cell states and the other toward Trm cells (**Figures 2H, I**). To further understand the biological characteristics of CD4^+^ T-cell clusters, we applied quantitative set analysis for gene expression (QuSAGE). The results revealed that the PPAR signaling pathway was predominantly enriched in Trm cells (Cluster 4) (**Supplementary Figure S3**).

### Colocalization of DEFA5 and aberrant CD4^+^ IELs activation

**Figure 3:**
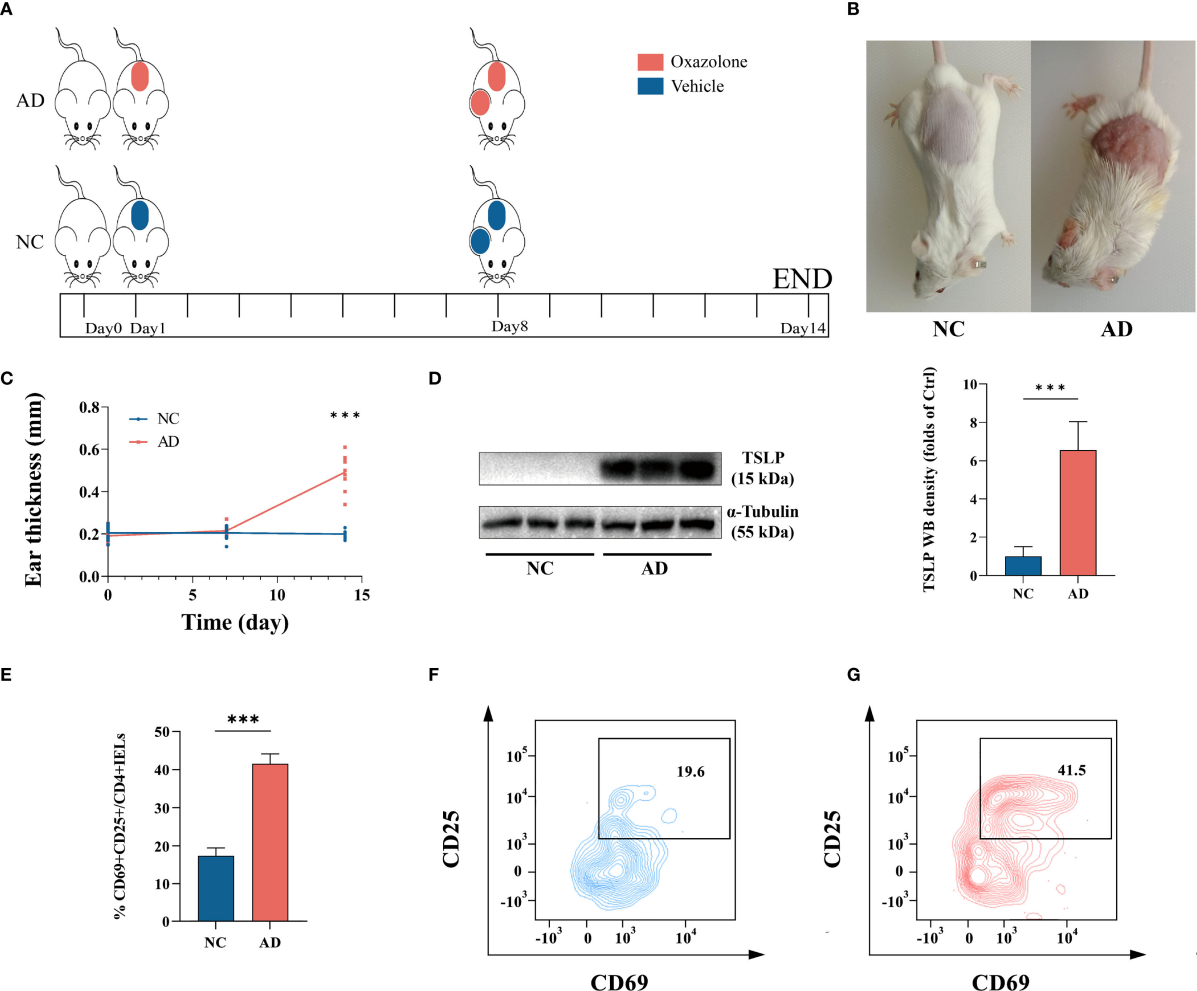
**(A)** The animal experimental protocol. **(B)** Representative images depicting atopic dermatitis-induced inflammatory lesions in a murine model. **(C)** Changes in mouse ear thickness were measured at three key points: once before prestimulation, once before ear stimulation, and once before tissue collection, ***p < 0.001 vs. NC. (n=10). **(D)** The protein levels of TSLP in ear tissue, as determined by western blotting, ***p < 0.001 vs. NC. (n=9). **(E–G)** Percentage of CD25+CD69+ expression within CD4+ IELs. ***p < 0.001 vs. NC. (n=3).

To further understand the underlying mechanisms of DEFA5 production by intestinal CD4^+^ T cells in AD, we established an oxazolone-mediated AD mouse model (**Figure 3A**). Compared with the control group, oxazolone-treated mice exhibited pronounced redness and swelling in both the ears and the back, along with a considerable increase in ear thickness (**Figures 3B, C**). Thymic stromal lymphopoietin (TSLP) is considered an a key marker that plays a significant role in advancing AD and is linked to gut microbiota imbalance related to the disease (41). Western blot analysis of TSLP showed increased levels of this protein in the ears of the treated mice compared to that in control group (**Figure 3D**), confirming the establishment of the AD in the mice. IELs constitute the most abundant lymphatic system within the intestine, and CD4^+^ T cells within IELs are predominantly located in the distal part of the small intestine (42). IELs are also considered to be one of the most abundant and intestine-specific subsets of Trm cells (43). Thus, we isolated single CD4+ IELs from the mouse intestine and detected them using confocal microscopy. The findings indicated that stimulated CD4+ IELs generated more DEFA5 than unstimulated CD4+ IELs, with CD4 and DEFA5 clearly colocalizing (**Supplementary Figure S4A**). In addition, flow cytometry results demonstrated a significant increase in CD25 and CD69 (**Figures 3E–G**) within the CD4^+^ IELs, indicating aberrant activation of CD4^+^ IELs in mice with AD.

### PPARγ regulates DEFA5 expression in CD4^+^ IELs

**Figure 4:**
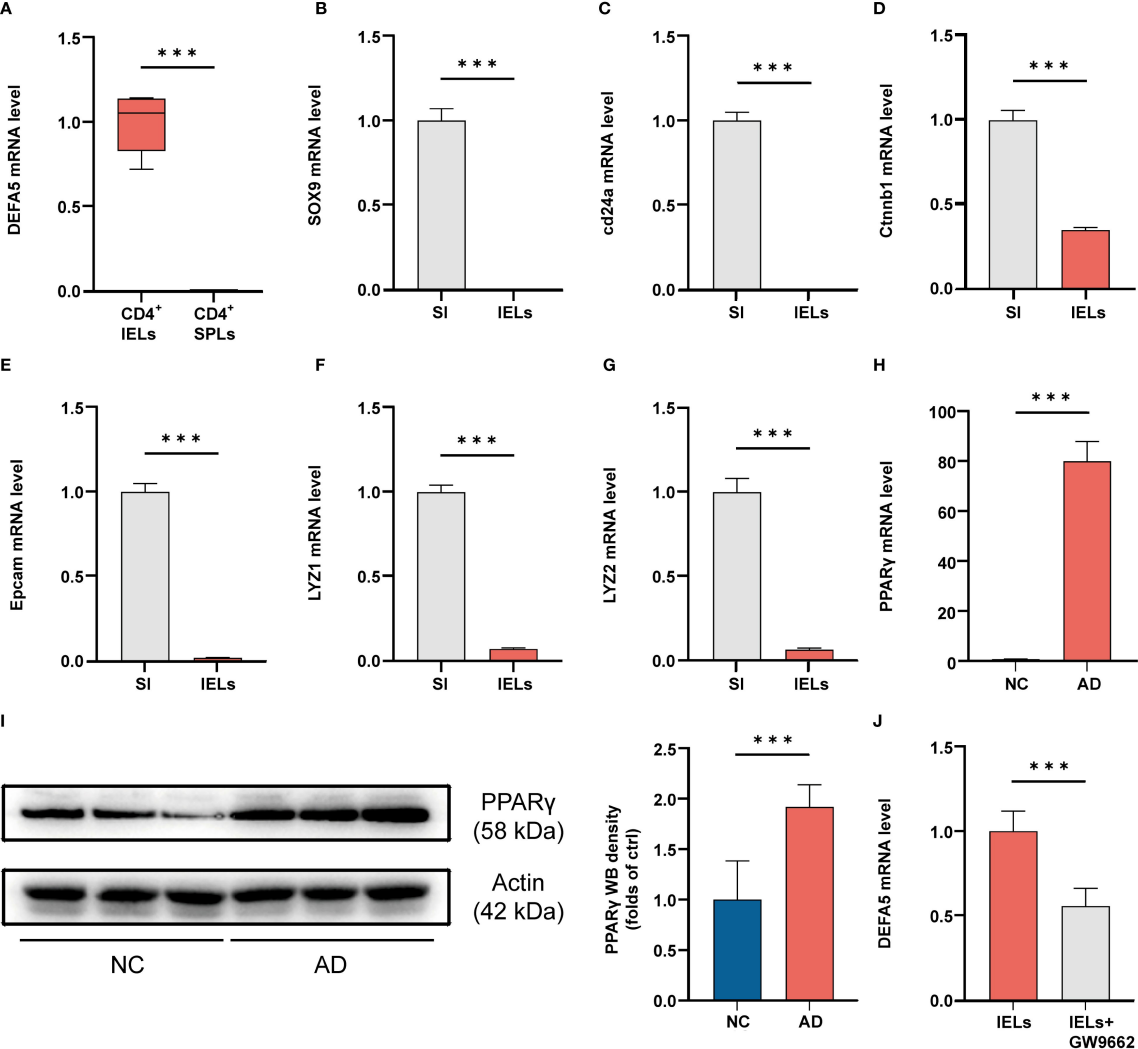
**(A)** After IELs and SPLs were isolated, the cells were stimulated and then collected for PCR analysis. ***p < 0.001 vs. IELs. (n=3). **(B-G)** Cells were collected from the entire small intestine (SI) and IELs, and then the mRNA levels of *SOX9*, *cd24a*, *ctnnb1*, *Epcam*, *Lyz1* and *Lyz2* were analyzed by PCR. ***p < 0.001 vs. SI. (n=3). **(H)** The mRNA levels of PPARγ in the ileum, as determined by polymerase chain reaction, ***p < 0.001 vs. NC. (n=3). **(I)** The protein levels of PPARγ in the ileum, as determined by western blotting, ***p < 0.001 vs. NC. (n=9). **(J)** After IELs were isolated, they were either stimulated with GW9662 (10 μM) or left unstimulated for 24 hours, followed by PCR analysis to assess the mRNA levels of *DEFA5* (n=3).

To test if activation could lead to DEFA5 expression in CD4^+^ T cells from other tissues, we evaluated *DEFA5* expression in CD4^+^ T cells from the spleen and IELs through PCR upon activation. Moreover, DEFA5 expression was markedly elevated in stimulated CD4^+^ IELs as opposed to unstimulated CD4^+^ IELs (**Supplementary Figure S4A**). The results showed that CD4^+^ IELs, but not CD4^+^ T cells from the spleen, were capable of expressing *DEFA5* (**Figure 4A**). Moreover, DEFA5 expression was markedly elevated in stimulated CD4^+^ IELs as opposed to unstimulated CD4^+^ IELs (**Supplementary Figure S4B**). In addition, epithelial cells, particularly Paneth cells, are recognized as key producers of DEFA5. To address potential contamination by Paneth and other epithelial cells, we examined the expression of Paneth cell-specific markers and epithelial cell-specific markers, including *Sox9*, *Epcam*, *ctnnb1*, *Lyz2*, *Lyz1*, and *Cd24a* in the isolated CD4^+^ IELs. The results showed that these genes were undetectable in the isolated IELs (**Figures 4B–G**), suggesting that the CD4^+^ IELs obtained were unlikely contaminated by Paneth cells or epithelial cells. Furthermore, flow cytometric evaluation of the purified CD4^+^ IELs indicated that 99.8% of the CD45^+^ cells were CD4^+^, showing minimal presence of non-immune or non-CD4^+^ T cells (**Supplementary Figure S5**).

Preliminary single-cell RNA sequencing (scRNA-seq) analyses have shown a significant upregulation of the PPAR signaling pathway in ADs. We therefore evaluated the expression of PPARγ in the mouse intestine. Compared to the control group, a notable increase in PPARγ mRNA and protein levels was observed in the intestines of the mice with AD (**Figures 4H, I**). Previous findings suggest that PPARγ activation plays a key role in preserving defensin expression within the intestine (44). To investigate whether PPARγ is capable of regulating DEFA5, we used AlphaFold3 to visualize the potential for a direct binding interface between DEFA5 and PPARγ (**Supplementary Figure S6A**). Furthermore, we utilized the GEPIA web tool (http://gepia.cancer-pku.cn/) to conduct a Spearman correlation analysis, aiming to investigate the link between DEFA5 and PPARG gene expression (**Supplementary Figure S6C**). The results demonstrated a significant positive correlation between PPARG and DEFA5 expression (R = 0.54, p < 0.001), which indicates that increased expression of PPARγ is frequently linked to the upregulation of DEFA5. Next, PPARγ inhibitor GW9662 was used to test the role of this molecule in DEFA5 expression by CD4^+^ IELs. The results showed that following the addition of GW9662, a significant decrease in *DEFA5* expression was observed compared to cells without PPARγ inhibition (**Figure 4J**), indicating that PPARγ plays an important role in regulation of DEFA5 expression by CD4^+^ IELs.

### CD4^+^ IELs contribute to AD-associated intestinal damage possibly through DEFA5

**Figure 5:**
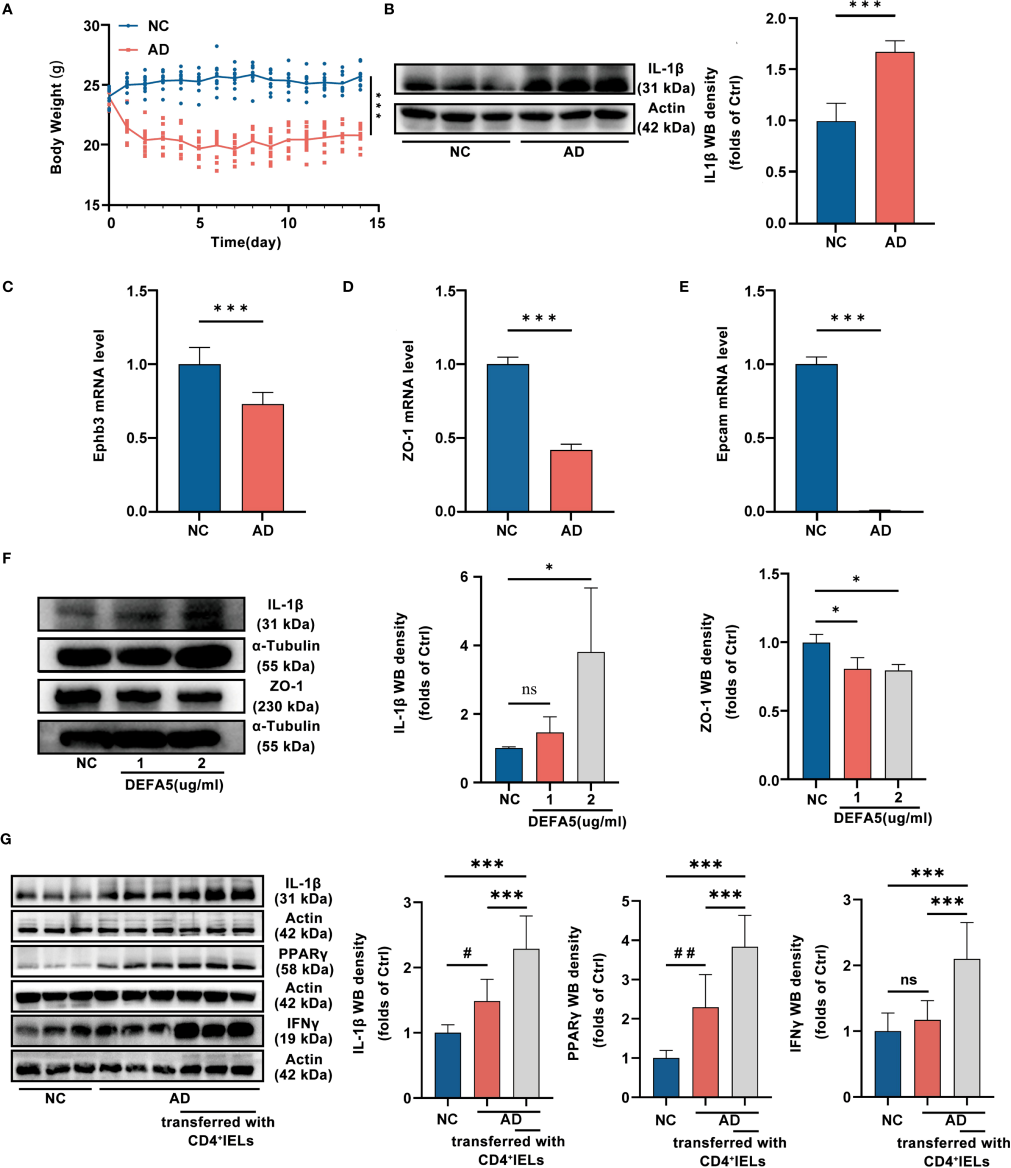
**(A)** Variations in mouse body weight over a 14-day feeding period. Statistical significance is indicated for all time points except the initial one. ***p < 0.001 vs. NC. (n=10). **(B)** The protein levels of IL-1β in the ileum, as determined by western blotting, ***p < 0.001 vs. NC. (n=9). **(C-E)** The mRNA levels of *Ephbe*, *Epcam*, and *ZO-1* in the ileum, as determined by PCR; ***p < 0.001 vs. NC. (n=3). **(F)** The protein levels of ZO-1 and IL-1β in the ileum, as determined by western blotting, *p < 0.05 vs. NC (n=9). **(G)** The protein levels of IL-1β, PPARγ and IFNγ in the ileum, as determined by western blotting, ##*p* < 0.01 vs. AD. #*p* < 0.05 vs. AD; ***p < 0.001 vs. The AD group that transferred with CD4^+^IELs. (n=9).

Upon induction of AD, we noted a significant reduction in body weight in the AD group of mice compared to the control group (**Figure 5A**). Additionally, we assessed the mRNA expression of *Epcam*, *Ephb3*, and *ZO-1*, as well as the protein levels of IL-1β in the intestine. It was discovered that mice with AD exhibited reduced expression of *Epcam*, *Ephb3*, and *ZO-1*, but increased expression of IL-1β compared to control (**Figures 5B–E**), suggesting the impairment of intestinal barrier function and the development of intestinal inflammation. Patients with ADs frequently experience gastrointestinal symptoms such as abdominal pain, bloating, and diarrhea (45, 46). This phenomenon has also been validated in various AD animal models (47). Under these circumstances, intestinal inflammation may lead to the aberrant activation of intestinal CD4^+^ T cells, resulting in the production of DEFA5. Paneth cells constitute the principal cellular source of DEFA5 in humans. Single-cell transcriptomic analysis revealed an increased abundance of Paneth cells in the ileum of individuals with AD (**Supplementary Figure S7A**). Additionally, within the intestinal epithelium, the expression of *DEFA5* in the ADs was significantly greater than that in the NCs (**Supplementary Figure S7B**). It has demonstrated that elevated local concentrations of DEFA5 are also among the factors that induce apoptosis (48). To test this, we treated the intestinal epithelial cell line HT-29 cells with 1 µg/ml and 2 µg/ml of DEFA5, followed by examination of ZO-1 and IL-1β by western blot. The results showed a dose-dependent reduction in ZO-1 expression and increase in IL-1β (**Figure 5F**), suggesting that DEFA5 may play important roles in the compromise of intestinal barrier function and the development of intestinal inflammation in AD.

To investigate the contribution of CD4^+^ IELs to the development of intestinal inflammation in mice with AD, we adoptively transferred CD4^+^ IELs into SCID mice with or without AD induction. Compared with NC group and AD group without T cell transfer, SCID mice that received CD4^+^ IELs exhibited significantly increased expression of PPARγ, accompanied by elevated levels of IL-1β and IFNγ (**Figure 5G**). Additionally, we co-cultured intestinal explants with either stimulated or unstimulated CD4^+^ IELs. ZO-1 expression in explants was significantly lower when co-cultured with stimulated CD4^+^ IELs than when co-cultured with unstimulated CD4^+^ IELs or without any CD4^+^ IEL co-culture (**Supplementary Figure S4C**). These results suggested that the abnormal increase of CD4^+^ IELs could be a key factor contributing to the development of intestinal inflammation in AD.

## Discussion

Previous research has indicated that in march of atopic dermatitis, AD often precedes other types of allergic diseases and is subsequently accompanied by various gastrointestinal symptoms (49, 50). Our findings suggested that AD-associated intestinal inflammation may be linked to changes in the functionality of intestinal immune cells. Based on single-cell transcriptomic analysis, we detected aberrant expression of *DEFA5* in intestinal CD4^+^ T cells. Further studies demonstrated that this aberrant *DEFA5* expression is unique to CD4^+^ IELs, as it is not found in CD4^+^ T cells from the spleen. In addition, PPARγ was found to be important for the expression of DEFA5 by the CD4^+^ IELs.

Recent studies have provided evidence of inflammation and impairment of the intestinal barrier in both mouse and canine models of AD (51, 52). Moreover, an investigation involving 4,175 participants has discerned that individuals afflicted with AD demonstrate an elevated prevalence of gastrointestinal disorders compared to the control group (53). Currently, studies on the mechanisms underlying intestinal inflammation in AD primarily focus on the gut microbiota (54). However, some studies have confirmed that microorganisms do not serve as mediators in the gastrointestinal inflammation associated with AD (55). Moreover, current scRNA-seq efforts concerning AD predominantly focus on the skin rather than the intestine (56, 57). Therefore, we aimed to elucidate the relationship between AD and gastrointestinal inflammation through the application of scRNA-seq. Consequently, we identified 22 cell clusters, including epithelial cell types, endothelial cells, T_NK cells, B-cell populations, plasma cells, macrophages, fibroblasts, and mast cells in the intestines of ADs. Considering the pivotal role of T cells in inflammation and defensin production, we annotated the transcriptomic features of T_NK cell clusters. Based on differential gene expression analysis, we observed a significant increase in *DEFA5* expression in T cells from ADs. Further investigation revealed an abnormal increase in *DEFA5* expression in the intestinal CD4^+^ T cells of these patients. Gene Ontology enrichment analysis indicated a close association between CD4^+^ T cells and defensin production. Previously, a scRNA-seq study on AD demonstrated that AD lesional skin, in contrast to normal skin, exhibited a more pronounced infiltration of CD4^+^ Trm cells (58). In this study, through pseudotime trajectory analysis and QuSAGE, we similarly observed an increased abundance of CD4^+^ Trm cells in the ADs compared to NCs in the intestine. Interestingly, in chronic intestinal inflammation, CD4^+^ Trm cells have been found to accelerate the progression of the disease (12). It is highly possible that CD4^+^ Trm cells contribute to the development of intestinal inflammation and impaired barrier function in AD patients.

CD4^+^ IELs are known to promote inflammation through the secretion of inflammatory factors (11). Using single CD4^+^ IELs isolated from the mouse intestine, we demonstrated the colocalization of CD4 and DEFA5. Moreover, stimulated CD4^+^ IELs exhibited significantly increased DEFA5 expression compared to unstimulated cells. Flow cytometry data showed that compared to NC group, CD4^+^ IELs from AD group exhibited significantly increased expression of CD69 and CD25. We considered the possibility that CD25^+^ cells might be regulatory T cells (Tregs), while CD25 is widely regarded as a marker of Tregs. However, previous studies have also shown that CD25 can indicate activation in T cells (59). In our study, we found that CD25 was co-expressed with CD69 (60, 61). This pattern suggests that these cells are likely activated CD4^+^ IELs rather than Tregs. Building on the scRNA-seq results, we further showed that CD4^+^ IELs, but not splenic CD4^+^ T cells, were capable of expressing DEFA5, contributing to an exacerbation of intestinal inflammation. PPAR family members have been shown to regulate T cell activation and differentiation (62). Consistently, we observed increased expression of PPARγ in SCID mice that were adoptively transferred with CD4^+^ IELs, along with upregulation of inflammatory cytokines.

Previous studies have suggested that PPARγ, a nuclear receptor, plays a role in mucosal defense regulation (63). For instance, components of gut microbiome, such as *Lactobacillus rhamnosus* I5007, have been shown to promote butyrate production and activate PPARγ, thereby enhancing defensin levels (64). Spatial transcriptomic analysis of sebaceous glands in AD has revealed a significant enrichment of the PPARγ gene (65), indicating its association with the disease. Our pathway analysis results showed upregulation of PPAR pathway in the intestine of AD (**Figure 2D**). Therefore, we hypothesize that the mechanism by which CD4^+^ T cells produce DEFA5 may related to PPARγ. To investigate the role of PPAR in regulation of DEFA5 expression, a PPARγ inhibitor (GW9662) was used to ascertain the regulatory role of PPARγ. The results confirmed the importance of this molecule in the expression of *DEFA5* in CD4^+^ IELs.

DEFA5 typically acts as a “protector” in the intestinal environment (66, 67). However, studies have also shown that DEFA5 is significantly increased in the terminal ileum of patients with ulcerative colitis compared with healthy controls (68). In addition, patients with higher DEFA5 levels were also found to be more likely to experience pouchitis recurrence (69). These studies indicate that DEFA5 could be a contributing factor to disease pathogenesis in certain conditions. In this study, we also demonstrated that treatment with DEFA5 led to a reduction in the expression of tight junction proteins and an increase in the expression of inflammatory factors. Therefore, the CD4^+^ IELs-mediated localized upregulation of DEFA5 may exacerbate inflammation in the intestine, potentially representing an important mechanism underlying gut injury in AD. Nevertheless, the precise mechanism by which DEFA5 contributes to intestinal inflammation has yet to be elucidated. We postulate that it may be associated with the recruitment of immune cells (8) and its effects on gut microbiota dynamics (70). Bioinformatics analysis coupled with machine learning techniques suggested that DEFA5 is a pivotal gene that is significantly correlated with the progression of ulcerative colitis (71). Consequently, DEFA5 holds promise as a prospective biomarker candidate for discerning the presence of intestinal damage in clinical presentations of AD. Nonetheless, substantiating this potential necessitates additional investigative endeavors. For instance, using CD4^+^ IELs from DEFA5 knockout mice or adoptively transferring CD4^+^ T cell populations incapable of producing DEFA5 as control groups would help further elucidate the specific contribution of DEFA5 to AD-associated intestinal inflammation. Moreover, despite single-cell transcriptome analysis revealing differential expression of *DEFA5* in terminal ileum tissue cells of patients with AD, the samples were limited, as only five patients were included in the healthy controls. Another limitation of this study is that we did not assess the TCR repertoire of DEFA5^+^ CD4^+^ T cells. Future studies incorporating TCR-seq would be valuable to clarify the antigen specificity and developmental trajectory of this cell subset.

In summary, utilizing a scRNA-seq approach, we identified aberrant gene expression in the terminal ileum tissues of ADs. In addition, a novel function of CD4^+^ IELs in the production of DEFA5 was discovered, indicating that CD4^+^ T lymphocytes may adopt new roles in the context of certain autoimmune diseases. Exploring the functions of these cells across various microenvironments could offer new perspectives for therapeutic strategies.

## Data Availability

The datasets presented in this study can be found in online repositories. The names of the repository/repositories and accession number(s) can be found below: GSE228148 (GSE).
