# Supplementary material for: DEFA5-producing CD4+ T cells in the intestines of atopic dermatitis patients play an important role in the development of AD-associated intestinal inflammation
Source: Front Immunol. 2025 Sep 19;16:1535527. doi: 10.3389/fimmu.2025.1535527 (PMC12491060; doi:10.3389/fimmu.2025.1535527)
Supplement: Supplementary file 1 [file SupplementaryFile1.docx]

Supplementary Material

## Supplementary Figures


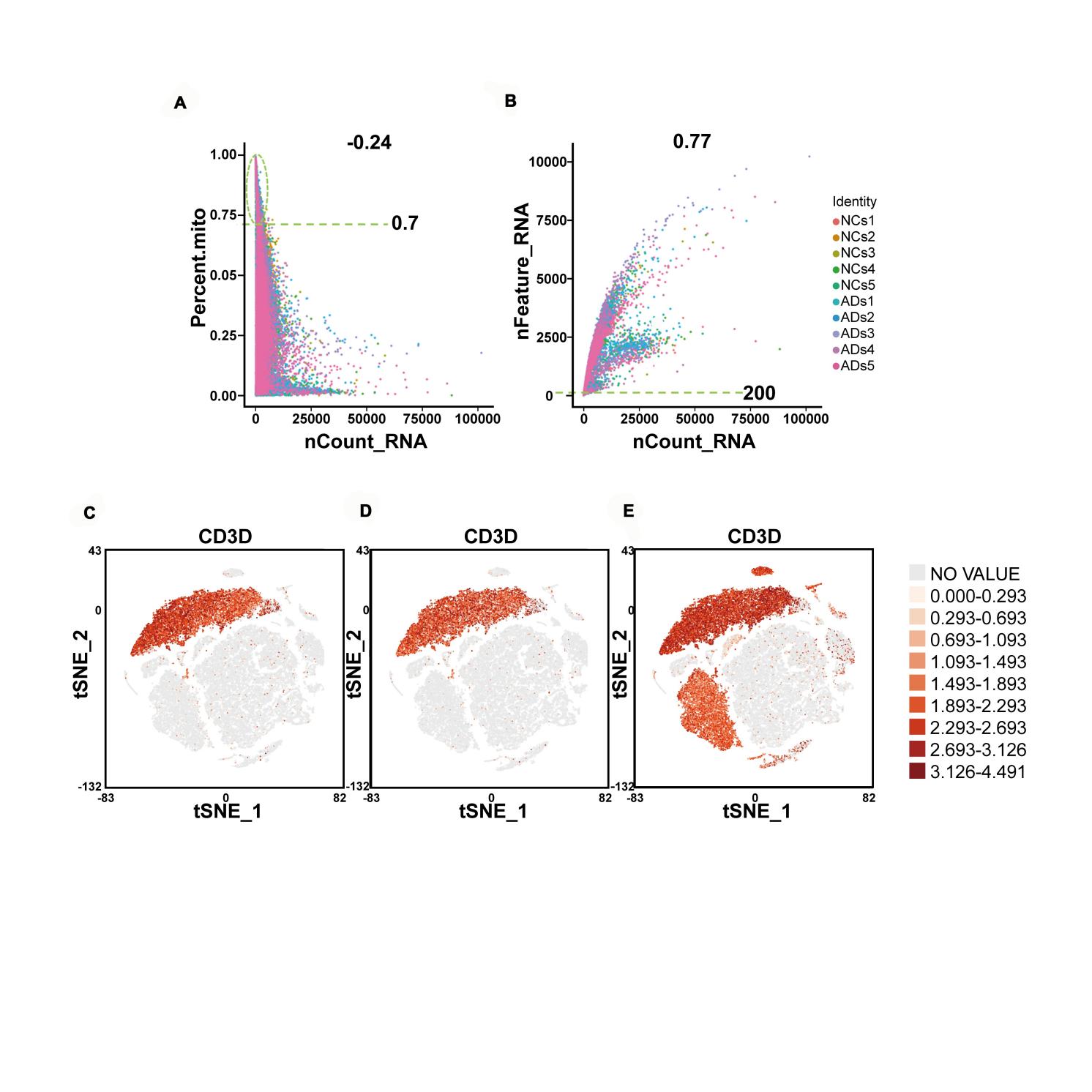


**Supplementary Figure 1.** **Quality control and Identification of T_NK**

(A)The relationship between the percentage of mitochondrial genes and the mRNA reads was assessed, with the filtering threshold set at 0.7. (B) The relationship between the amount of mRNA and the mRNA reads. Cells exhibiting fewer than 200 detected genes were excluded from further analysis. (C-E) tSNE color-coded for expression (gray to red) of key cell-type markers to define the T_NK cells.

**
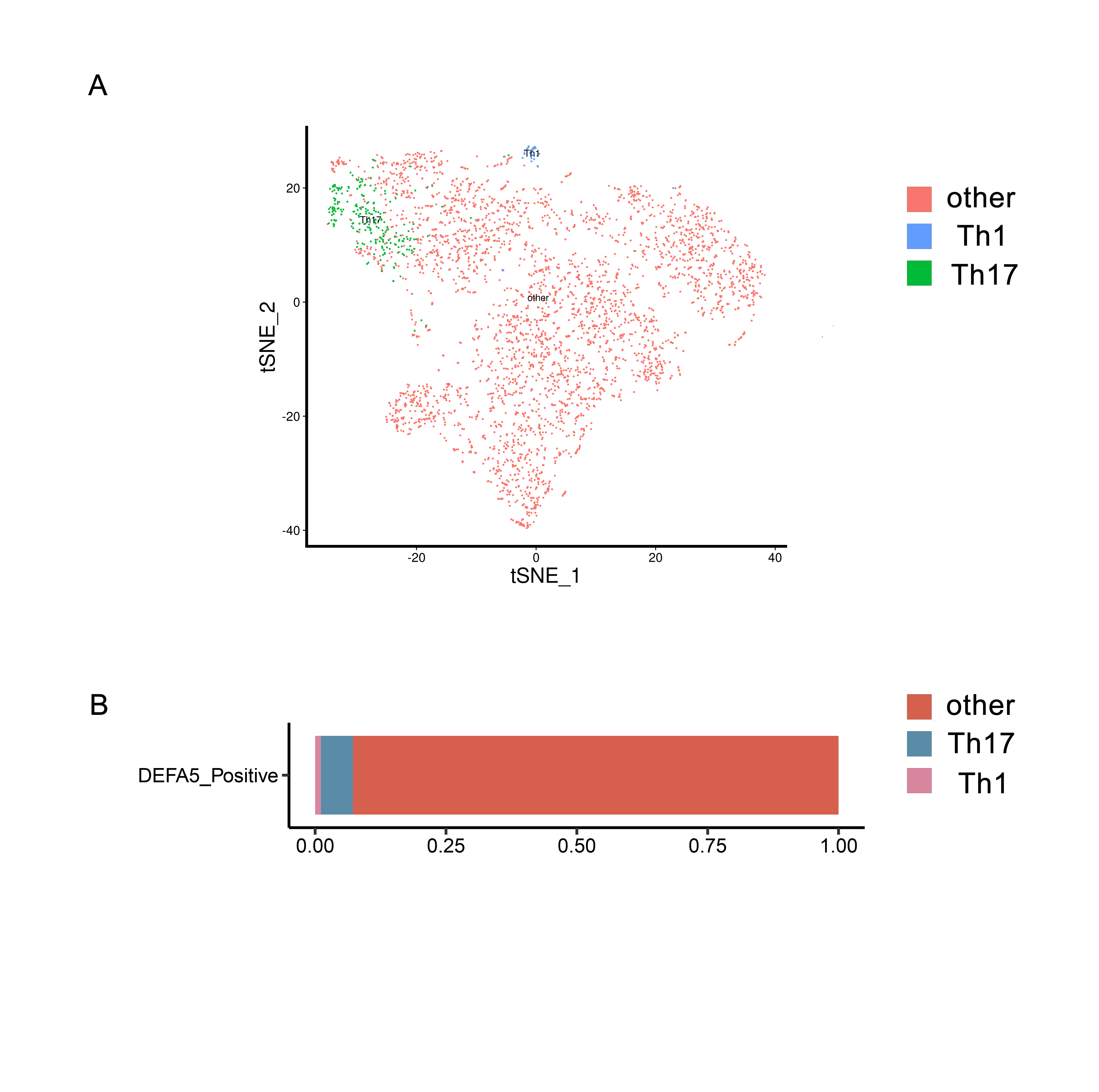
**

**Supplementary Figure 2.** **Characterization of CD4^+^ DEFA5^+^ T cell subsets**

(A) t-SNE of DEFA5^+^CD4^+^ T cells redefined into three subcellular groups (Th1, Th17, and other cells). (B) Proportional distribution of different subsets within DEFA5^+^ CD4^+^ T cell populations (Th1, Th17, and other cells).


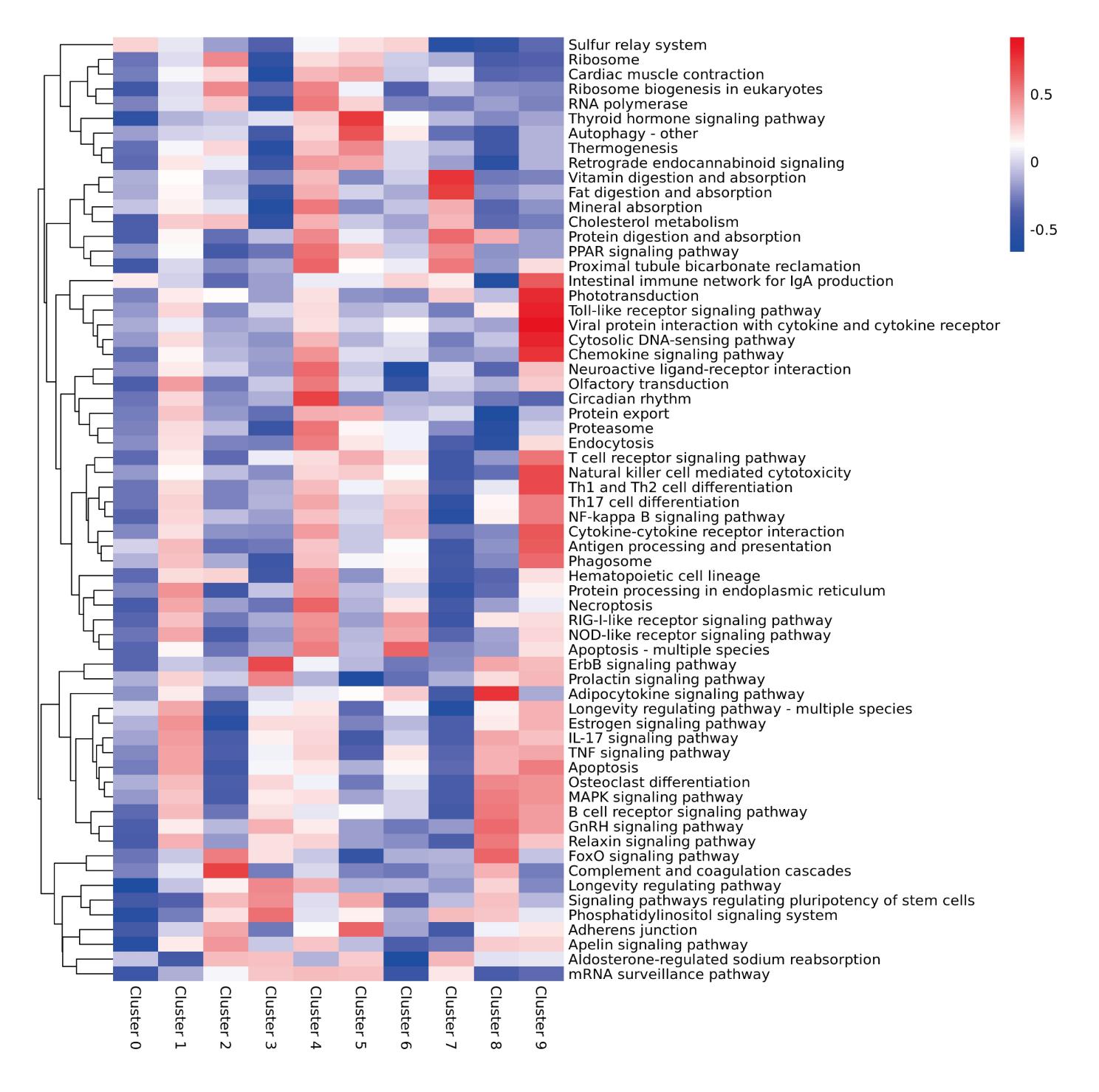


**Supplementary Figure 3. Quantitative Set Analysis for Gene Expression in CD4+ T Cells**

The significance of each cluster within CD4+ T cells across various gene sets is indicated by color: closer to red signifies higher significance, while closer to blue denotes lower significance.


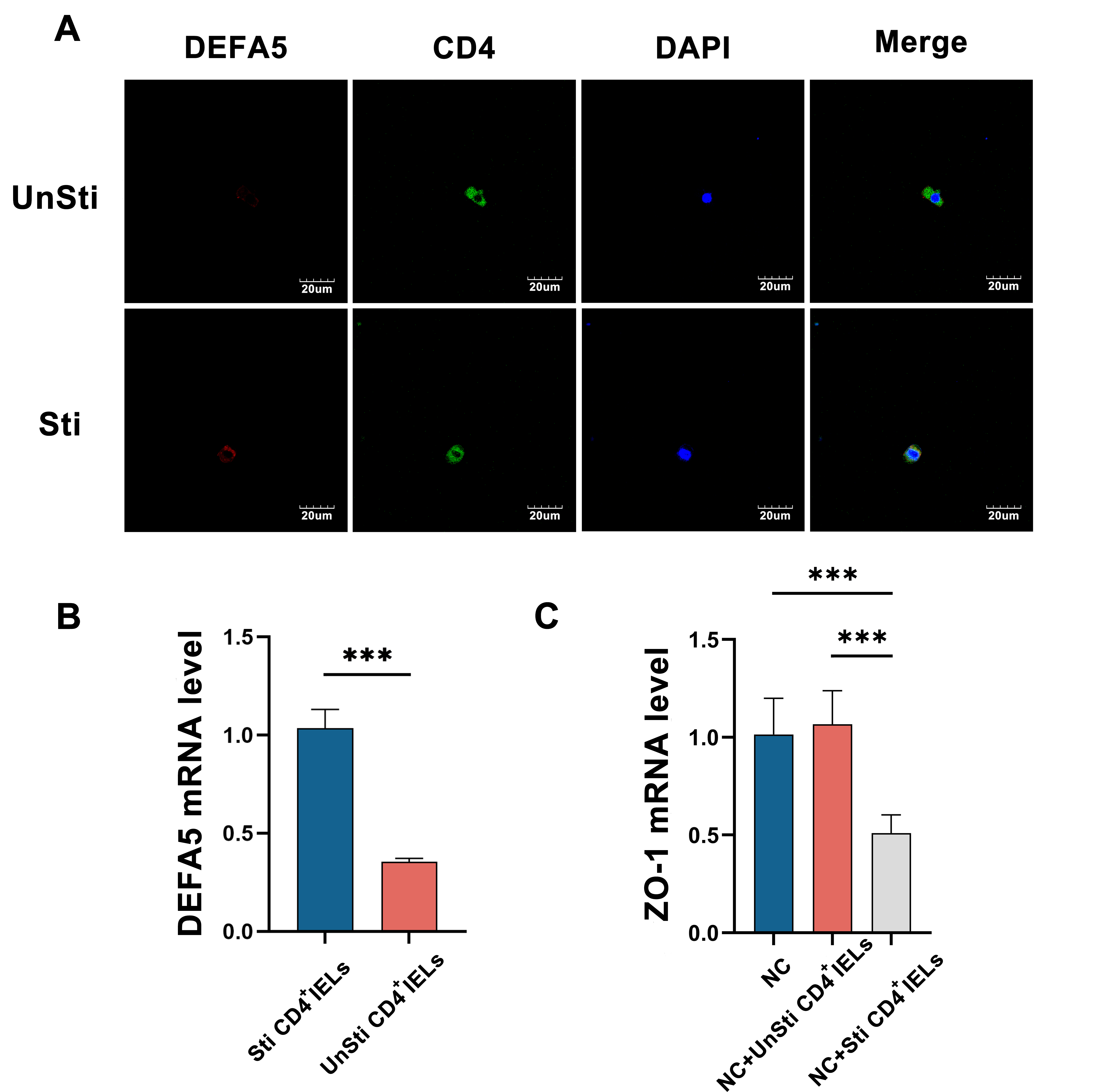


**Supplementary Figure 4.**

1. Immunofluorescence staining of DEFA5 in isolated CD4^+^ IELs. Representative images of CD4^+^ IELs from Unstimulated (Unsti) and Stimulated (Sti) groups stained for DEFA5 (red), CD4 (green), and nuclei (DAPI, blue). Scale bar: 20 μm. (B) After CD4^+^ IELs were isolated, cells were either left unstimulated (Unsti) or stimulated (Sti), and then collected for PCR analysis of DEFA5 mRNA expression. ***p < 0.001 vs. Sti CD4^+^ IELs. **p < 0.01 vs. Sti CD4^+^ IELs. *p < 0.05 vs. Sti CD4^+^ IELs (n = 9). (C) After intestinal explants were co-cultured with or without CD4^+^ IELs, samples were collected for PCR analysis of ZO-1 mRNA expression. NC: explants cultured alone without any treatment; NC+UnSti CD4^+^ IELs: explants co-cultured with unstimulated CD4^+^ IELs; NC+Sti CD4^+^ IELs: explants co-cultured with stimulated CD4^+^ IELs. ***p < 0.001 vs. NC. **p < 0.01 vs. NC. *p < 0.05 vs. NC (n = 9).


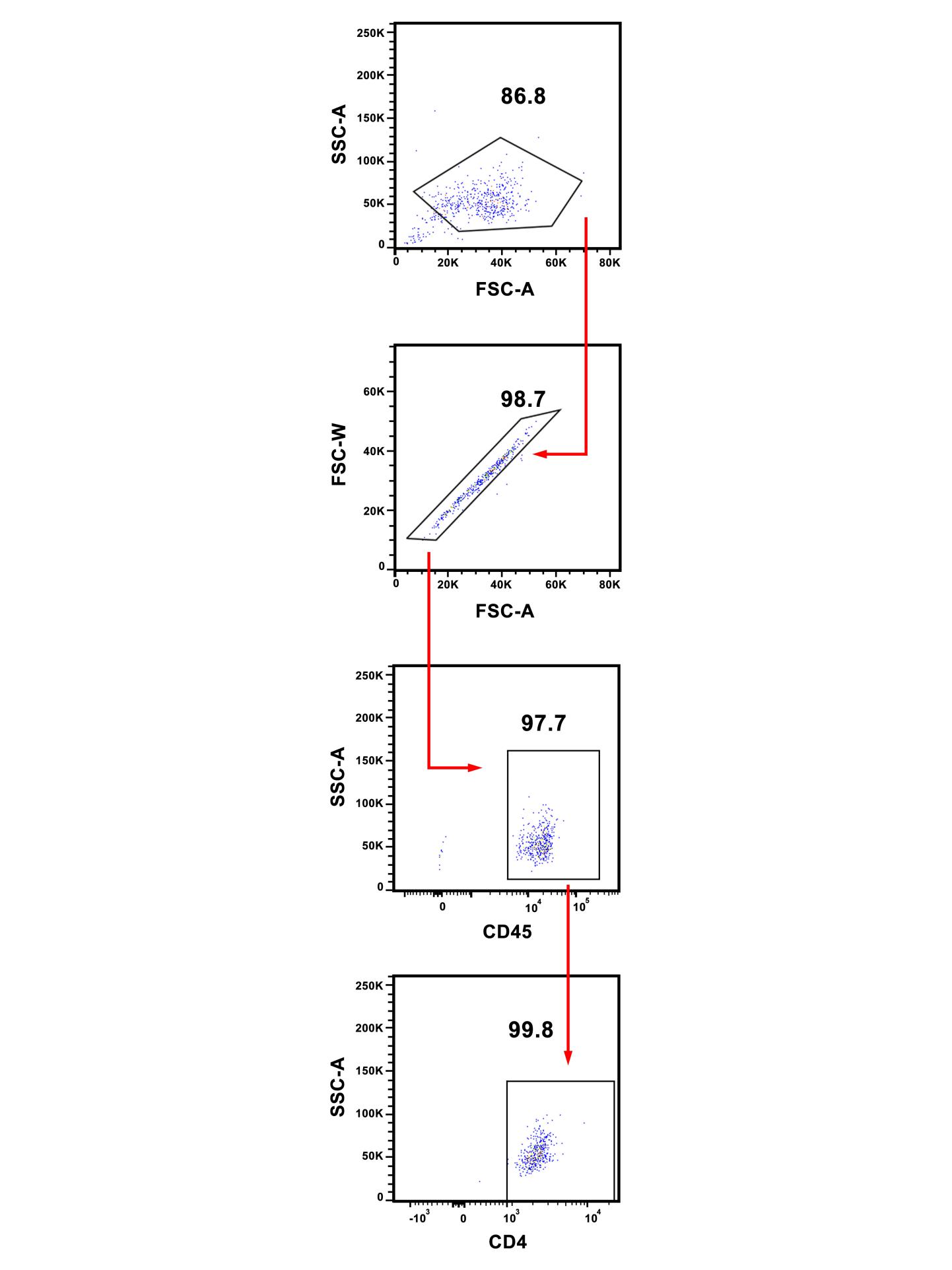


**Supplementary Figure 5. Flow cytometry was performed on purified CD4^+^ IELs to assess cell purity. Red arrow indicates the order for gating.**

**
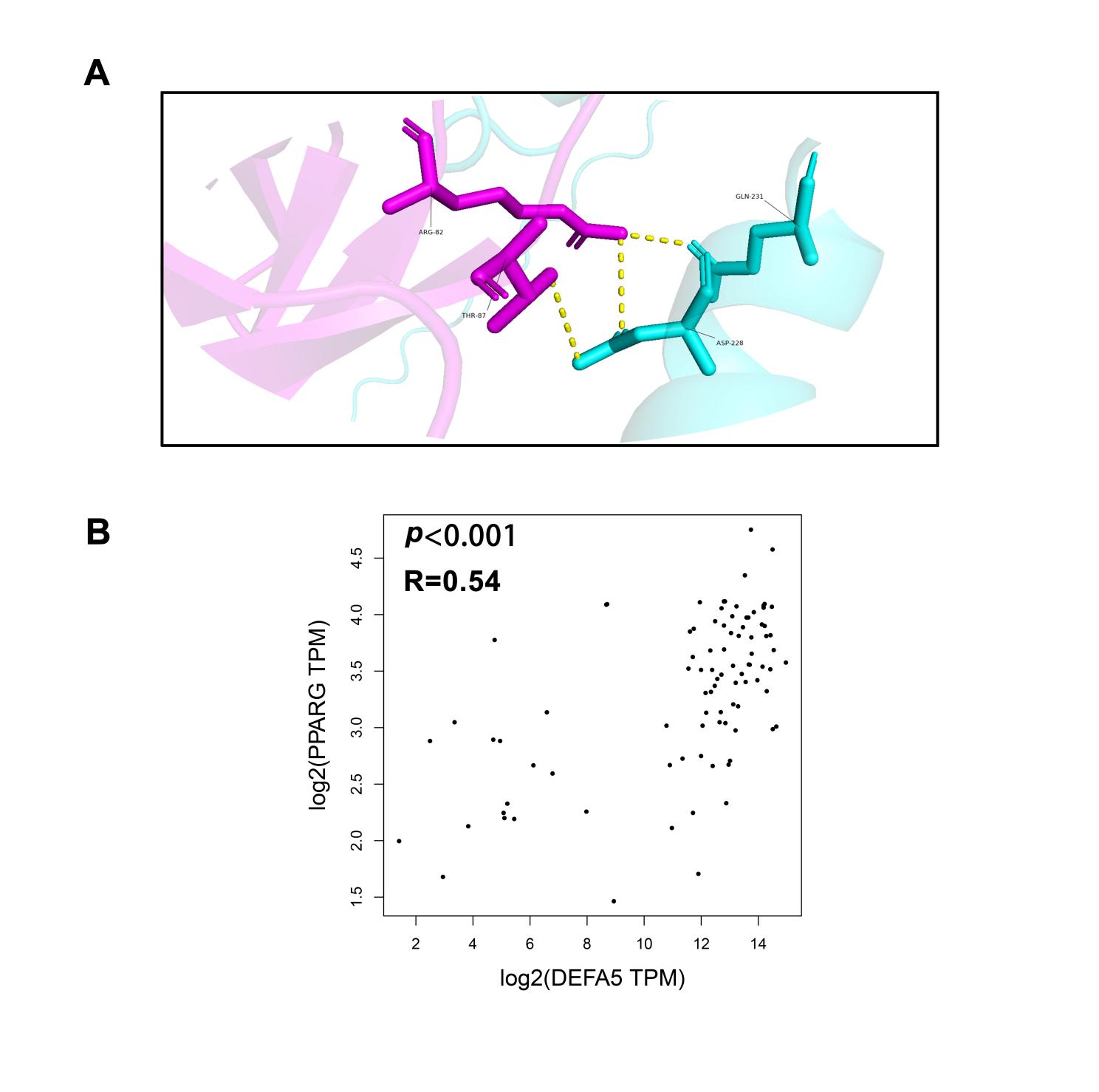
**

**Supplementary Figure 6.**

1. Structural model of DEFA5 and PPARγ predicted by AlphaFold3. The potential interaction interface is highlighted, suggesting a possible direct interaction between the two proteins. (B) Spearman correlation between PPARG and DEFA5 expression levels (R = 0.54, p = 0.000000023).


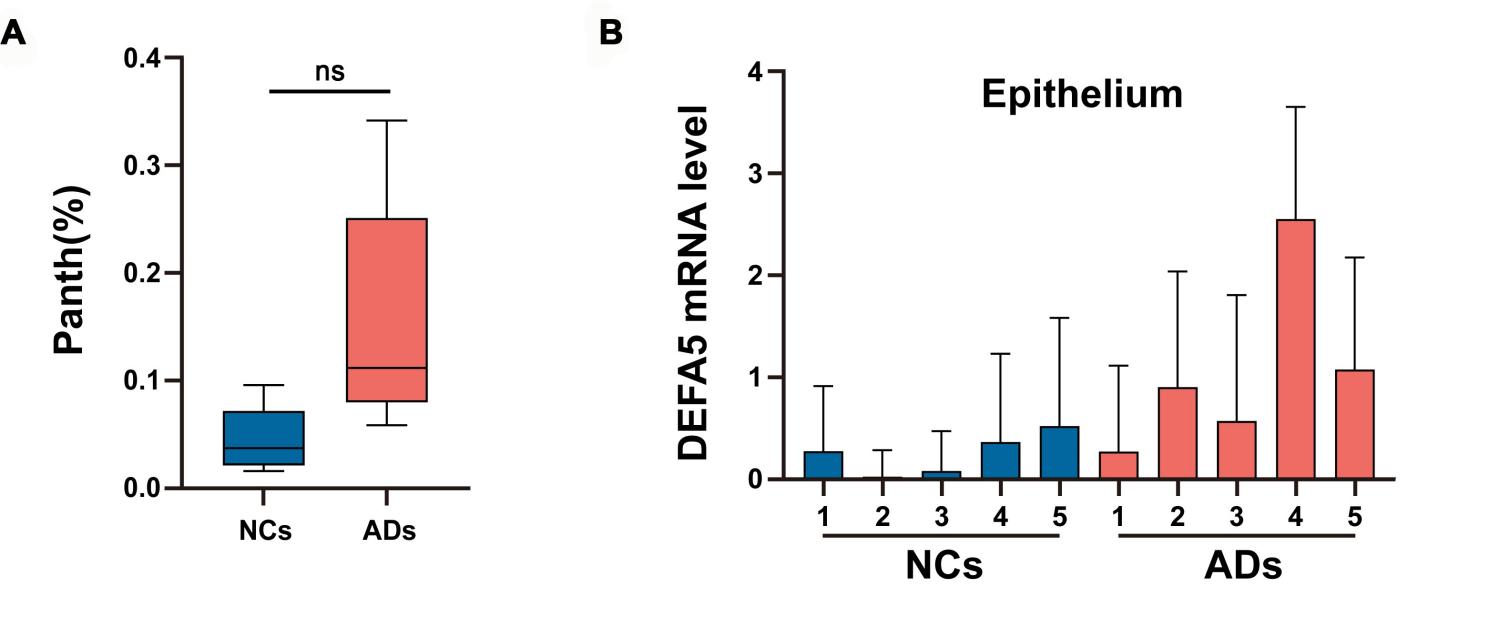


**Supplementary Figure 7. Paneth Cell Counts and DEFA5 Expression in the Epithelium**

(A) Box plot illustrating the percentage of Paneth cells in the NC (blue) and AD (red) samples. (B) Bar charts displaying the expression levels of DEFA5 in epithelial cells from individual donors.
