# Supplementary material for: DEFA5-producing CD4+ T cells in the intestines of atopic dermatitis patients play an important role in the development of AD-associated intestinal inflammation
Source: Front Immunol. 2025 Sep 19;16:1535527. doi: 10.3389/fimmu.2025.1535527 (PMC12491060; doi:10.3389/fimmu.2025.1535527)
Supplement: Supplementary file 2 [file Table1.docx]

**Supplementary Table 1. Clinical information of donors for scRNAseq.**

| Donor ID | Age | Gender | Race | Tissue site | Examination |
| --- | --- | --- | --- | --- | --- |
| NC1 | 38 | male | yellow | terminal ileum | enteroscopy |
| NC2 | 43 | female | yellow | terminal ileum | enteroscopy |
| NC3 | 26 | female | yellow | terminal ileum | enteroscopy |
| NC4 | 26 | male | yellow | terminal ileum | enteroscopy |
| NC5 | 28 | female | yellow | terminal ileum | enteroscopy |
| AD1 | 54 | male | yellow | terminal ileum | enteroscopy |
| AD2 | 24 | male | yellow | terminal ileum | enteroscopy |
| AD3 | 31 | female | yellow | terminal ileum | enteroscopy |
| AD4 | 24 | female | yellow | terminal ileum | enteroscopy |
| AD5 | 49 | female | yellow | terminal ileum | enteroscopy |
