# Supplementary material for: DEFA5-producing CD4+ T cells in the intestines of atopic dermatitis patients play an important role in the development of AD-associated intestinal inflammation
Source: Front Immunol. 2025 Sep 19;16:1535527. doi: 10.3389/fimmu.2025.1535527 (PMC12491060; doi:10.3389/fimmu.2025.1535527)
Supplement: Supplementary file 3 [file Table2.docx]

| Reagent | Catalog number | Brand | RRID |
| --- | --- | --- | --- |
| FITC-Anti-Mouse CD3e | 553061 | BD Pharmingen | AB_394594 |
| BV421-Anti-Mouse CD4 | 562891 | BD Pharmingen | AB_2737870 |
| PE-Anti-Mouse CD25 | 553075 | BD Pharmingen | AB_394605 |
| BV510-Anti-Mouse CD69 | 563030 | BD Pharmingen | AB_2737963 |
| TSLP Polyclonal antibody | 13778-1-AP | Proteintech | AB_2208528 |
| Alpha Tubulin Polyclonal antibody | 11224-1-AP | Proteintech | AB_2210206 |
| PPAR Gamma Polyclonal antibody | 16643-1-AP | Proteintech | AB_10596794 |
| IL-1 Beta Polyclonal antibody | 16806-1-AP | Proteintech | AB_10646432 |
| Alpha Actin Polyclonal antibody | 23660-1-AP | Proteintech | AB_2879307 |
| HRP-conjugated Affinipure Goat Anti-Rabbit IgG(H+L) | SA00001-2 | Proteintech | AB_2722564 |
| Defensin Alpha 5 | abx176113 | Abbexa | AB_3096419 |
| CD4 Monoclonal antibody | 67786-1-Ig | Proteintech | AB_2918550 |
| IFN gamma Polyclonal antibody | 28722-1-AP | Proteintech | AB_3086082 |
| CoraLite488-conjugated Goat Anti-Mouse IgG(H+L) | SA00013-1 | Proteintech | AB_2810983 |
| CoraLite594 – conjugated Goat Anti-Rabbit IgG(H+L) | SA00013-4 | Proteintech | AB_2810984 |

**Supplementary Table 2. Reagents Utilized in the Article**
