# Supplementary material for: DEFA5-producing CD4+ T cells in the intestines of atopic dermatitis patients play an important role in the development of AD-associated intestinal inflammation
Source: Front Immunol. 2025 Sep 19;16:1535527. doi: 10.3389/fimmu.2025.1535527 (PMC12491060; doi:10.3389/fimmu.2025.1535527)
Supplement: Supplementary file 4 [file Table3.docx]

| Gene | Forward | Reverse |
| --- | --- | --- |
| Mouse DEFA5 | TGTCCTCCTCTCTGCCCTTGTC | CTGGCTGCTCCTCAGTATTAGTCTC |
| Mouse SOX9 | GATCTGAAGAAGGAGAGCGAGGAAG | CACCAGCGTCCAGTCGTAGC |
| Mouse cd24a | GCTTCTGGCACTGCTCCTACC | CCTCTGGTGGTAGCGTTACTTGG |
| Mouse Ctnnb1 | GGAACGCAGCAGCAGTTTGTG | CCGAGCAAGGATGTGGAGAGC |
| Mouse Epacam | AAGAGCATGGACCTGAGAGTGAAC | GCTAATGACACCACCACAATGACAG |
| Mouse LYZ1 | GCAAAGAGGGTGGTGAGAGATCC | TCAGACTCCGCAGTTCCGAATATAC |
| Mouse LYZ2 | GCAAAGAGGGTGGTGAGAGATCC | TCAGACTCCGCAGTTCCGAATATAC |
| Mouse PPARG | TGTTCGCCAAGGTGCTCCAG | AAGGCTCATGTCTGTCTCTGTCTTC |
| Mouse Ephb3 | TACGGCTCAATGACGGACAGTTC | AGGATGTTTCGGGCAGCAAGG |
| Mouse ZO-1 | ACCCGAAACTGATGCTGTGGATAG | GCTGGCTGGCTGTACTGTGAG |

**Supplementary Table 3. Polymerase chain reaction primer sequences**
